# Efficacy of extracorporeal cardiopulmonary resuscitation compared to conventional cardiopulmonary resuscitation for adult cardiac arrest patients: a systematic review and meta-analysis

**DOI:** 10.1038/srep34208

**Published:** 2016-09-23

**Authors:** Chiwon Ahn, Wonhee Kim, Youngsuk Cho, Kyu-Sun Choi, Bo-Hyoung Jang, Tae Ho Lim

**Affiliations:** 1 Department of Emergency Medicine, College of Medicine, Hanyang University, Seoul, Korea; 2 Department of Emergency Medicine, College of Medicine, Hallym University, Seoul, Korea; 3 Department of Neurosurgery, College of Medicine, Hanyang University, Seoul, Korea; 4 Department of Preventive Medicine, College of Korean Medicine, Kyung Hee University, Seoul, Korea

## Abstract

We performed a meta-analysis to compare the impact of extracorporeal cardiopulmonary resuscitation (ECPR) to that of conventional cardiopulmonary resuscitation (CCPR) in adult patients who experience cardiac arrest of cardiac origin. A literature search was performed using criteria set forth in a predefined protocol. Report inclusion criteria were that ECPR was compared to CCPR in adult patients with cardiac arrest of cardiac origin, and that survival and neurological outcome data were available. Exclusion criteria were reports describing non-cardiac origin arrest, review articles, editorials, and nonhuman studies. The efficacies of ECPR and CCPR were compared in terms of survival and neurological outcome. A total of 38,160 patients from 7 studies were ultimately included. ECPR showed similar survival (odds ratio [OR] 2.26, 95% confidence interval [CI] 0.45–11.20) and neurologic outcomes (OR 3.14, 95% CI 0.66–14.85) to CCPR in out-of-hospital cardiac arrest patients. For in-hospital cardiac arrest (IHCA) patients, however, ECPR was associated with significantly better survival (OR 2.40, 95% CI 1.44–3.98) and neurologic outcomes (OR 2.63, 95% CI 1.38–5.02) than CCPR. Hence, ECPR may be more effective than CCPR as an adjuvant therapy for survival and neurologic outcome in cardiac-origin IHCA patients.

Cardiopulmonary resuscitation (CPR) is performed in adult cardiac arrest patients to elicit subsequent return of spontaneous circulation (ROSC) and recovery of cardiovascular and neurologic function^1^,^2^. Recent trends show that the survival rates of adult cardiac arrest patients are rising, and that these improvements are accompanied by more favourable neurological outcomes^3^. However, such advances remain far from satisfactory.

To increase the survival rate post-CPR, the application of extracorporeal membrane oxygenation (ECMO) during CPR, referred to as extracorporeal CPR (ECPR), has been selectively attempted on adult patients who experience cardiac arrest due to reversible aetiologies^4^,^5^,^6^,^7^. Recently, several successful ECPR cases have been reported in in-hospital cardiac arrest (IHCA) events, or have been witnessed in out-of-hospital cardiac arrest (OHCA) patients^4^,^8^,^9^,^10^,^11^,^12^,^13^,^14^,^15^,^16^,^17^.

ECPR is currently used as an adjuvant therapy of conventional cardiopulmonary resuscitation (CCPR). ECPR is not only a highly invasive technique but also requires an expert team approach^18^; nevertheless, it might be a suitable alternative for adult cardiac arrest patients in whom CCPR does not produce stable ROSC or is refractory to CPR.

This study aimed to evaluate the efficacy of ECPR compared to CCPR as an adjuvant therapy in adult cardiac-origin arrest patients. Hence, we performed a meta-analysis to examine survival rates and neurologic outcomes in ECPR and CCPR patients.

## Results

### Study and patient characteristics

**Figure 1:**
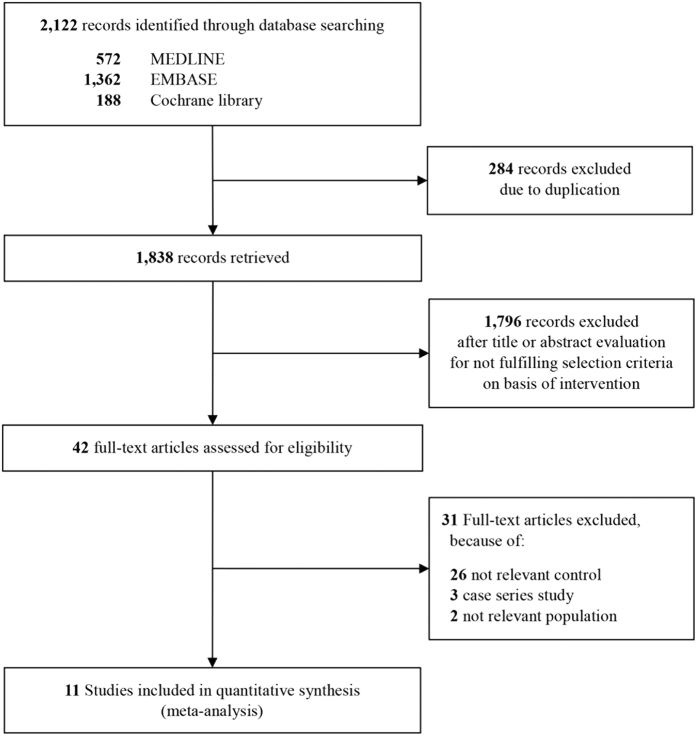
Flow chart of the study selection process for this meta-analysis.

Our literature search revealed 7 eligible studies; 38,160 patients were included in the meta-analysis (Fig. 1). The main attributes of these studies are shown in Table 1. All included studies were observational. Three studies described prospective patient cohorts and the remaining 4 described retrospective cohorts. Propensity score matching analysis was used in 5 studies. Overall, survival and neurologic outcomes were obtained from 7 and 6 articles, respectively. One study measured adjusted odds ratios (ORs) with 95% confidence intervals (CIs) using multivariate analysis, while 6 only compared the frequency of patients between survivors (good neurologic outcome) and non-survivors (poor neurologic outcome). In only 1 study were propensity score matching and multivariate ORs all used to adjust for covariates.

### Quality of the included studies

Among the 7 included studies, 3 fulfilled all of the quality criteria^4^,^11^,^16^ while 2 studies were assessed as high-risk owing to confounding variables^12^,^14^. When we were not able to assess quality because of the lack of a clear description of the contents of each risk of bias (ROB) domain, we described the quality as ‘unclear’. Five studies (2 on OHCA and 3 on IHCA) that used propensity score matching achieved >9 points in quality assessment and were therefore deemed high quality^4^,^9^,^11^,^16^,^17^. The other 2 studies were considered low quality^12^,^14^ (see Supplementary Figs S1 and S2).

### Clinical endpoints

#### Survival in ECPR vs. CCPR recipients

**Figure 2:**
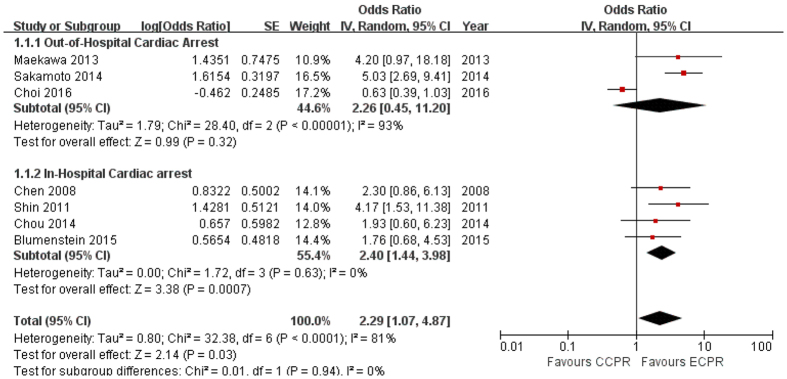
Survival to discharge from hospital or to 28 days post-cardiac arrest. CI: confidence interval, SE: standard error.

Although the overall survival rate was higher in ECPR than CCPR (OR 2.29, 95% CI 1.07–4.87), ECPR showed significantly better survival compared to CCPR only in IHCA patients (OR 2.40, 95% CI 1.44–3.98; I^2^ = 0%). However, the survival rate in ECPR recipients was similar to that of CCPR recipients among OHCA patients (OR 2.26, 95% CI 0.45–11.20; I^2^ = 93%) (Fig. 2).

#### Neurologic outcome in ECPR vs. CCPR recipients

**Figure 3:**
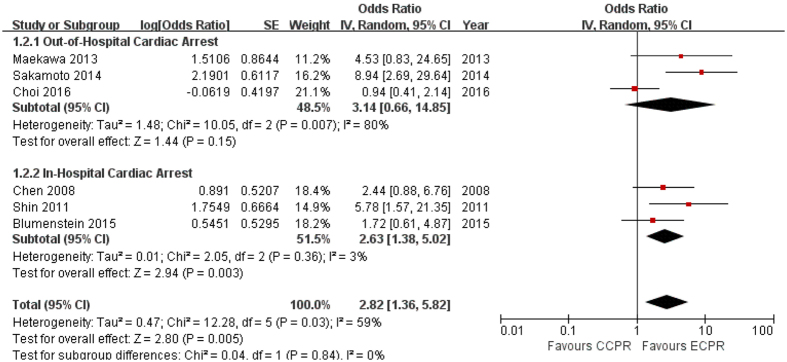
Good neurologic outcome (Cerebral Performance Category 1–2 or Glasgow Outcome Scale 1) to discharge from hospital, or for 28 or 90 days post-cardiac arrest. CI: confidence interval, SE: standard error.

Our accumulated data suggested that the overall neurologic outcome in ECPR patients was better than in CCPR patients (OR 2.82, 95% CI 1.36–5.82). ECPR showed similar neurologic outcome to CCPR in OHCA patients (OR 3.14, 95% CI 0.66–14.85; I^2^ = 80%), while ECPR was associated with better neurologic outcome than CCPR in IHCA patients (OR 2.63, 95% CI 1.38–5.02; I^2^ = 3%) (Fig. 3).

#### Subgroup analysis and sources of heterogeneity

We performed subgroup analysis according to the study type (prospective vs. retrospective), the assessment of quality, and the use of propensity score matching analysis in OHCA and IHCA patients.

Additionally, we analysed subgroups according to the presumed aetiology of arrest, initial electrocardiographic rhythm, and whether the arrest was witnessed.

##### Subgroup analysis in OHCA patients

ECPR was associated with better survival than CCPR when evaluated according to study type (OR 4.89, 95% CI 2.75–8.70; I^2^ = 0%). When assessing for quality, the survival owing to ECPR was similar to that owing to CCPR (OR 1.43, 95% CI 0.23–9.00), and heterogeneity was still high (I^2^ = 83%).

The neurologic outcome in ECPR recipients was better than in CCPR recipients when analysed according to study type (OR 7.12, 95% CI 2.68–18.95; I^2^ = 0%). However, ECPR patients showed similar neurologic outcomes to CCPR patients when assessing for quality (OR 1.72, 95% CI 0.38–7.71; I^2^ = 63%) (Table 2).

##### Subgroup analysis in IHCA patients

ECPR was associated with better survival than CCPR when analysed according to study type (OR 2.44, 95% CI 1.35–4.41; I^2^ = 0%). When assessing for quality, ECPR was significantly associated with better survival than CCPR (OR 2.52, 95% CI 1.44–4.43) with low heterogeneity (I^2^ = 0%).

The neurologic outcome in ECPR recipients was also better than in CCPR recipients when analysed for study type (OR 2.95, 95% CI 0.19–9.59; I^2^ = 51%) and for assessment of quality (OR 2.63, 95% CI 1.38–5.02; I^2^ = 3%) (Table 2).

##### Subgroup analysis according to additional factors

ECPR showed similar survival to CCPR with high heterogeneity when analysing patients with arrests of presumed cardiac origin (OR 1.62, 95% CI 0.64–4.09; I^2^ = 73%). In patients where the aetiologies of cardiac arrest were not stated, ECPR was associated with better survival than CCPR (OR 3.54, 95% CI 1.89–6.64; I^2^ = 40%). Only 1 study specifically described initial ventricular fibrillation or pulseless ventricular tachycardia in terms of initial electrocardiographic rhythm; in the remaining 6 studies, the survival rates owing to ECPR and CCPR were similar (OR 1.92, 95% CI 0.92–3.98; I^2^ = 73%).

ECPR was associated with better neurologic outcome than CCPR patients in reports not mentioning the aetiologies of arrest (OR 4.27, 95% CI 1.53–11.89; I^2^ = 56%) or the nature of the initial electrocardiogram rhythm (OR 2.15, 95% CI 1.12–4.14; I^2^ = 42%).

All studies that limited their analyses to witnessed arrests were in IHCA patients; in this group, ECPR was associated with better survival and neurologic outcomes than CCPR, with low heterogeneity (survival OR 2.40, 95% CI 1.44–3.98; I^2^ = 0%; neurologic outcome OR 2.63, 95% CI 1.38–5.02; I^2^ = 3%) (Table 3).

## Discussion

In this meta-analysis, we found that ECPR was associated with improved survival and neurologic outcome compared to CCPR in adult cardiac-origin IHCA patients. In contrast, ECPR showed survival and neurologic outcomes that were similar to CCPR in OHCA patients.

We excluded four studies after full-text review^8^,^10^,^13^,^14^. The reasons were as follows: First, Lin *et al.*^8^ and Shin *et al.*^10^ were excluded because their data sources were reproduced from Chen *et al.*^4^ and Shin *et al.*^9^ respectively. Second, Choi *et al.*^16^ and Kim *et al.*^13^ appear to have reported data from the same patients, as the same national South Korean database that held all data from OHCAs managed by the national Emergency Medical Services system had been used in both studies. Therefore, the study by Kim *et al.*^13^ that was performed at a single tertiary hospital was excluded. Third, the study by Siao *et al.*^15^ was also not included in the meta-analysis because OHCA and IHCA patient outcomes (such as survival or neurologic outcomes) were not reported separately.

This study excluded patients older than 75 years because aging is itself a significant variable in decreased survival^13^. Nevertheless, it is highly likely that aging is still a potent confounder for survival in our study because elderly patients under 75 years were included. Our analysis showed that the mean age of CCPR patients was higher than that of the ECPR group. Thus, unless propensity score matching was performed, the confounding effect of age could have resulted in the underestimation of survival and neurologic outcome in the CCPR group. The more elderly patients were included, the lower the survival rate achieved. In the meta-analysis of observational studies using score matching, the survival and neurologic outcomes of the ECPR group were significantly higher than that of the CCPR group only in IHCA patients (Table 2).

Because there are few randomized controlled trials in the field of resuscitation, several confounding factors should be controlled using multivariate analysis for the included observational studies. When performing our meta-analysis, we prioritized the adjusted ORs produced by multivariate analysis after score matching. Unfortunately, certain articles provided only univariate ORs after score matching; hence, the score for confounding factors was necessarily low upon evaluation for ROB. It should be noted that the score matching method cannot completely eliminate selection bias in the included studies. Moreover, survival to discharge data is subject to significant influence by actions such as withdrawing life-sustaining therapy, prolonging life support in ECPR candidates, or abiding by do-not-resuscitate orders.

In this study, heterogeneities in the forests plots are indicative of major confounding factors. Therefore, a random effect model was employed for statistical analysis because of ECPR’s inconsistent effect on outcome. Nevertheless, there was some selection bias in the included studies because their recruitment periods spanned more than a decade, during which technological changes such as the emergence of mechanical CPR devices occurred and CPR guidelines were updated twice (in 2005 and 2010). Some included studies also reported combined outcomes, including ECPR with therapeutic hypothermia or coronary interventions, while others either did not combine outcomes or did not specify whether or not they did, making comparisons difficult.

In subgroup analysis for both IHCA and OHCA patients, ECPR was associated with better survival and neurologic outcome than CCPR in IHCA patients. Additionally, heterogeneity was low in studies for IHCA patients, illustrating consistency among the results for these patients. We postulate that the association of ECPR with better survival rates may be due to cardiac events in IHCA patients being detected more promptly by clinicians; ECPR could be selectively implemented in patients deemed to have reversible cardiac arrest causes after application of ECMO.

In contrast, ECPR and CCPR produced almost equal survival rates and neurologic outcomes in OHCA patients; furthermore, heterogeneity was high among OHCA patients, revealing inconsistency among the results. This was likely because survival of ECPR recipients among OHCA patients was affected by prehospitalization factors such as delay before intervention and prehospital CPR quality^19^. Compared to IHCA patients, the resolution of the cardiac-based causes for OHCA patients could therefore have been delayed^20^.

On the other hand, post-resuscitation care for OHCA patients, including both reperfusion therapy and therapeutic hypothermia, should be considered in the evaluation of the effectiveness of ECPR. One study reported that there was no significant survival benefit in ECPR compared to CCPR after score matching for post-resuscitation care^16^. This suggests that post-resuscitation care could be the most important factor in survival and neurologic outcomes regardless of ECMO use during CPR.

In a recent systematic review^21^, the benefit of ECPR was undetectable when compared to CCPR. Nevertheless, our analysis incorporated an additional, critical meta-analysis that included 36,180 patients^16^; as a result, we showed that ECPR was significantly more beneficial than CCPR in cardiac origin IHCA patients.

There were several limitations in this study. First, complications due to ECPR that could affect the survival and neurologic outcome of patients were not reported in the included studies. In particular, bleeding after ECPR is considered an important complication^22^,^23^,^24^,^25^,^26^. In the 2014 ELSO registry, incidences of significant bleeding of an internal organ such as the central nervous system (2.2%) or gastrointestinal tract (4.0%) were reported after ECPR, in addition to minor bleeding in the cannular (19.8%) or surgical sites (23%). Moreover, life-threatening complications of ECPR such as cardiac tamponade (5.4%) and disseminated intravascular coagulation (4.1%) were reported^26^.

Second, scoring for the severity of the cause of arrest was not reported in the included studies. Successful rescue angioplasty guarantees the survival of patients after ROSC^27^,^28^,^29^. In contrast, the patients do not have a high chance of survival after failed rescue angioplasty, even after undergoing ECPR. The failure of coronary angioplasty correlates with the increase in both the number of obstructed coronary vessels and the severity of each coronary artery obstruction. In patients with triple vessel disease, heart surgery may be required^30^,^31^. Similarly, the severity score is closely associated with survival and neurologic outcome.

Third, generalizability is not ensured in this study because the included patients were geographically confined to East Asia (Taiwan, Japan and South Korea)^4^,^9^,^11^,^12^,^13^,^16^. Therefore, our results may have been different if cohorts from other countries or races were included. Only 2 of the included studies were multicentre investigations^12^,^16^, while the remainder were all single-centre investigations^4^,^9^,^11^,^13^. Along with race, the low number of multicentre studies also contributed to decreased representation. To yield more robust conclusions, additional studies that have wide representation are required.

Fourth, logistics and human resources that are strongly correlated with the survival rate of CPR are not reported in the included studies. Time for ambulance arrival and time to first shock by automated external defibrillators are well-known CPR-related parameters. However, there are few studies reporting such logistics in our systematic review^11^,^16^. Factors such as the willingness of the lay public to perform CPR and training of hospital teams are also not evident in our included studies.

In conclusion, data suggest that ECPR is more effective than CCPR as an adjuvant therapy for survival and neurologic outcome in cardiac-origin IHCA patients. However, no such benefits were observed for ECPR in OHCA patients.

## Methods

Our study was based on the principles outlined by the Meta-analysis of Observational Studies in Epidemiology (MOOSE)^32^ and the Preferred Reporting Items for Systematic Reviews and Meta-analysis (PRISMA) groups^33^. Briefly, we devised a question based on population, intervention, comparison, and outcome (PICO). To that end, literature searches and critical assessments were performed. We summarized the eligible studies, and their outcomes were evaluated in a meta-analysis. The PICO question was as follows: “In adult patients of cardiac-origin arrest (P), does cardiopulmonary resuscitation with ECMO (I), compared to conventional cardiopulmonary resuscitation (C), improve survival rate and neurological outcome (O)?”

### Search strategy

A literature search was performed by 2 experienced reviewers (C. Ahn and W. Kim) on 22 December 2015. The search encompassed the MEDLINE and EMBASE databases via the Ovid interface, as well as the Cochrane library. Search terms included “cardiopulmonary resuscitation” or “resuscitation” or “heart massage” or “out-of-hospital cardiac arrest” or “cardiac arrest” or “cardiac massage” or “CPR” and “extracorporeal circulation” or “extracorporeal membrane oxygenation” or “extracorporeal oxygenation” or “ECMO” or “E-CPR” or “ECPR” or “ECLS” or “extracorporeal life support”. We included articles that reported any prospective or retrospective cohort studies that addressed our PICO question.

### Study selection

All identified studies were inputted into the reference management software Endnote 7.4. The 2 reviewers checked the title, abstract, or type of each of the identified articles. We did not consider articles that fell under the following exclusion criteria: reviews, case reports, editorials, letters, comments, conference abstracts, or meta-analyses; animal studies; languages other than English; duplicate studies; irrelevant populations; and inappropriate controls. In case of disagreement between the 2 reviewers, a third reviewer (KS Choi) intervened, and differences were discussed until consensus was reached. After eliminating the excluded abstracts, we acquired the full-texts of the chosen articles, which were then rescreened and evaluated more thoroughly for eligibility using the same exclusion criteria. Ultimately, our selected studies included adult patients (age 18–75 years) who received CCPR or ECPR after cardiac arrest due to cardiac origin.

### Risk of bias in individual studies

The Risk of Bias Assessment Tool for Nonrandomized Studies was used to assess ROB in our included studies^34^. ROB assessment was performed across 6 domains: selection of participants, confounding variables, measurement of exposure, blinding of outcome assessment, incomplete outcome data, and selective reporting. If there was disagreement of opinion in the ROB assessment, the majority decision was accepted. We used the following 3 quality criteria to assess ROB from the standpoint of confounding variables: the use of multivariate ORs, observational studies that are prospective in nature, and the use of propensity score matching. If at least 2 out of 3 criteria were fulfilled, the ROB was assessed as low risk. Otherwise, the ROB was assessed as high risk.

The methodological scores of identified studies were assigned values of 2, 1, and 0 for low, unclear, and high-risk studies, respectively. Studies achieving more than 9 points after totalling each 6-domain score were considered to be high quality.

### Outcome measures

The outcome was defined as survival and neurological outcome at hospital discharge or thereafter. The neurological outcome scores were dichotomized as good or poor based on the Glasgow Outcome Scale (1: good outcome; 2–5: poor outcome) and Cerebral Performance Category Scale (1–2: good outcome; 3–5: poor outcome).

### Statistical analysis

We combined studies using the Review Manager software version 5.3 (RevMan; The Cochrane Collaboration 2012, The Nordic Cochrane Centre, Copenhagen, Denmark). Because the effect of ECPR in comparison with CCPR on survival or neurologic outcome was inconsistent, we used a random-effects model. We employed the generic inverse variance method in RevMan to estimate the average treatment effect (using OR) for each outcome, and measured the 95% CI. We also calculated 95% prediction intervals to estimate the range of plausible treatment effects. The heterogeneity in each analysis was quantified by tau-squared and I-squared statistics. We assessed the effect of ECPR treatment compared to CCPR, and performed a subgroup analysis based on the location of cardiac arrest (OHCA/IHCA), study type, and the use of propensity score matching.

## Additional Information

**How to cite this article**: Ahn, C. *et al.* Efficacy of extracorporeal cardiopulmonary resuscitation compared to conventional cardiopulmonary resuscitation for adult cardiac arrest patients: a systematic review and meta-analysis. *Sci. Rep.* **6**, 34208; doi: 10.1038/srep34208 (2016).

## Supplementary Material

Supplementary Information

## Figures and Tables

**Table 1:** Details of included studies.

Study	Recruitment period and country	Study type and place of cardiac arrest	Propensity score matching used	OR	Number of participants (ECPR/CCPR)	Age, mean (year)^a^		Outcome	
						ECPR	CCPR	Survival	Neurologic outcome (good/poor)
Chen^4^	2004–2006 Taiwan	Prospective Single centre IHCA	Yes	Univariate	172 (59/113)	57.4 ± 12.5	60.3 ± 13.3	discharge	CPC (12/345) discharge
Lin^8^ ^b^	2004–2006 Taiwan	Prospective Single centre IHCA	Yes	Univariate	118 (55/63)	59.0 ± 11.7	60.6 ± 12.7	28 days	CPC (12/345) discharge
Shin^9^	2003–2009 South Korea	Retrospective Single centre IHCA	Yes	Univariate	406 (85/321)	59.9 ± 15.3	61.6 ± 14.2	in hospital	GOS (1/2345) discharge
Shin^10^ ^b^	2003–2009 South Korea	Retrospective Single centre IHCA	Yes	Univariate	406 (85/321)	59.9 ± 15.3	61.6 ± 14.2	28 days	GOS (1/2345) 2 year
Maekawa^11^	2000–2004 Japan	Prospective Single centre OHCA	Yes	Univariate	162 (53/109	54 (47–60)	71 (59–80)	discharge	CPC (12/345) 90 days
Sakamoto^12^	2008–2012 Japan	Prospective Multi-centre OHCA	No	Univariate	454 (260/194)	56.3 ± N/A	58.1 ± N/A	28 days	CPC (12/345) 28 days
Kim^13^ ^c^	2006–2013 Korea	Prospective Single centre OHCA	Yes	Univariate	499 (55/444)	53 (41–68)	69 (56–77)	discharge	CPC (12/345) discharge
Chou^14^	2006–2010 Taiwan	Retrospective IHCA Single centre	No	Univariate	66 (43/23)	60.5 ± 11.6	69.6 ± 13.3	discharge	—
Siao^15^ ^d^	2011–2013 Taiwan	Retrospective Single centre IHCA	No	Multivariate	60 (20/40)	54.5 ± 11.9	60.2 ± 11.2	discharge	CPC (12/34) discharge
Blumenstein^17^	2009–2013 Germany	Retrospective Single centre IHCA	Yes	Univariate	353 (52/272)	72 (55–77.9)	75.29 (67.4–79.1)	30 days	CPC (12/345) 30 days
Choi^16^	2009–2013 South Korea	Retrospective Multi-centre OHCA	Yes	Multivariate	36,547 (320/36,227)	56 (45–68)	67 (54–77)	discharge	CPC (12/345) discharge

*Abbreviations:* OR, odds ratio; ECPR, extracorporeal cardiopulmonary resuscitation; CCPR, conventional cardiopulmonary resuscitation; IHCA, in-hospital cardiac arrest; OHCA, out-of-hospital cardiac arrest; CPC, Cerebral Performance Category Scale; GOS, Glasgow Outcome Scale; N/A, not available.

^a^Age was presented as median (interquartile range) or mean ± standard deviation.

^b^Not included in the meta-analysis because of duplicated data sources.

^c^Not included in the meta-analysis because the patient data was previously published in the Choi *et al.*^16^ trial.

^d^Not included in the meta-analysis because the patient data was not reported separately for OHCA and IHCA patients.

**Table 2:** Subgroup analysis according to the type of arrest (OHCA vs. IHCA).

		Survival				Neurologic outcomes			
Characteristics		*N*	OR (95% CI)	*p* value for heterogeneity	I^2^, %	*N*	OR (95% CI)	*p* value for heterogeneity	I^2^, %
OHCA	All	3	2.26 (0.45–11.20)	<0.00001	93	3	3.14 (0.66–14.85)	0.007	80
	Study type								
	ROS	1	0.63 (0.39–1.03)	N/A		1	0.94 (0.41–2.14)	N/A	
	POS	2	4.89 (2.75–8.70)	<0.00001	0	2	7.12 (2.68–18.95)	0.52	0
	Assessment of quality								
	High*	2	1.43 (0.23–9.00)	0.02	83	2	1.72 (0.38–7.71)	0.10	63
	Low	1	5.03 (2.69–9.41)	N/A		1	8.94 (2.69–29.64)	N/A	
IHCA	All	4	2.40 (1.44–3.98)	0.63	0	3	2.63 (1.38–5.02)	0.36	3
	Study type								
	ROS	3	2.44 (1.35–4.41)	0.43	0	2	2.95 (0.19–9.59)	0.16	51
	POS	1	2.30 (0.86–6.13)	N/A		1	2.44 (0.88–6.76)	N/A	
	Assessment of quality								
	High*	3	2.52 (1.44–4.43)	0.46	0	3	2.63 (1.38–5.02)	0.36	3
	Low	1	1.93 (0.60–6.23)	N/A	—	—		—	

*Abbreviations: N*, the number of studies; OR, odds ratio; 95% CI, 95% confident interval; OHCA, out-of-hospital cardiac arrest; IHCA, in-hospital cardiac arrest; ROS, retrospective observational study; POS, prospective observational study; N/A, not available.

*High-quality studies were those that achieved >9 points in quality assessment. The propensity score matching method was used in all high-quality studies.

**Table 3:** Subgroup analysis of studies according to presumed aetiology, initial ECG rhythm, and whether the arrest was witnessed.

Characteristics	Survival				Neurologic outcomes			
	*N*	OR (95% CI)	*p* value for heterogeneity	I^2^, %	*N*	OR (95% CI)	*p* value for heterogeneity	I^2^, %
All	7	2.29 (1.07–4.87)	<0.0001	81	6	2.82 (1.36–5.82)	0.03	59
Presumed aetiology								
Cardiac-origin	4	1.62 (0.64–4.09)	0.01	73	3	1.81 (0.75–4.32)	0.16	46
Not stated	3	3.54 (1.89–6.64)	0.19	40	3	4.27 (1.53–11.89)	0.10	56
Initial ECG rhythm								
VF/pulseless VT	1	5.03 (2.69–9.41)	N/A		1	8.94 (2.69–29.64)	N/A	
Not stated	6	1.92 (0.92–3.98)	0.003	73	5	2.15 (1.12–4.14)	0.14	42
Witnessed arrest								
Witnessed*	4	2.40 (1.44–3.98)	0.63	0	3	2.63 (1.38–5.02)	0.36	3
Not stated	3	2.26 (0.45–11.20)	<0.00001	93	3	3.14 (0.66–14.85)	0.007	80

*Abbreviations: N*, the number of studies; OR, odds ratio; 95% CI, 95% confident interval; ECG, electrocardiogram; VF, ventricular fibrillation; VT, ventricular tachycardia; N/A, not available.
