# Supplementary material for: Efficacy of extracorporeal cardiopulmonary resuscitation compared to conventional cardiopulmonary resuscitation for adult cardiac arrest patients: a systematic review and meta-analysis
Source: Sci Rep. 2016 Sep 23;6:34208. doi: 10.1038/srep34208 (PMC5034223; doi:10.1038/srep34208)
Supplement: Supplementary Information [file srep34208-s1.pdf]

Efficacy of extracorporeal cardiopulmonary resuscitation compared to conventional cardiopulmonary resuscitation for adult cardiac arrest patients: a systematic review and meta-analysis

Chiwon Ahn[1], Wonhee Kim[2*], Youngsuk Cho[2], Kyu-Sun Choi[3], Bo-Hyoung Jang[4], Tae Ho Lim[1]

[1]Department of Emergency Medicine, College of Medicine, Hanyang University, Seoul, Korea

[2]Department of Emergency Medicine, College of Medicine, Hallym University, Seoul, Korea

[3]Department of Neurosurgery, College of Medicine, Hanyang University, Seoul, Korea

[4]Department of Preventive Medicine, College of Korean Medicine, Kyung Hee University, Seoul, Korea

***Corresponding author:** Wonhee Kim, M.D.

Department of Emergency Medicine, College of Medicine, Hallym University, Seoul, Korea

1, Singil-ro, Yeongdeungpo-gu, Seoul, 07441, Korea

Tel: +82-2-929-5561

Fax: +82-2-842-4217

E-mail: wonsee02@gmail.com

Word count: 2,894

This research was supported by the Civil research projects for solving social problems through the National Research Foundation of Korea (NRF) funded by the Ministry of Science, ICT, and Future Planning (NRF-2015M3C8A7A02027410).

Supplementary Figure 1 Graph of risk of bias for this meta-analysis

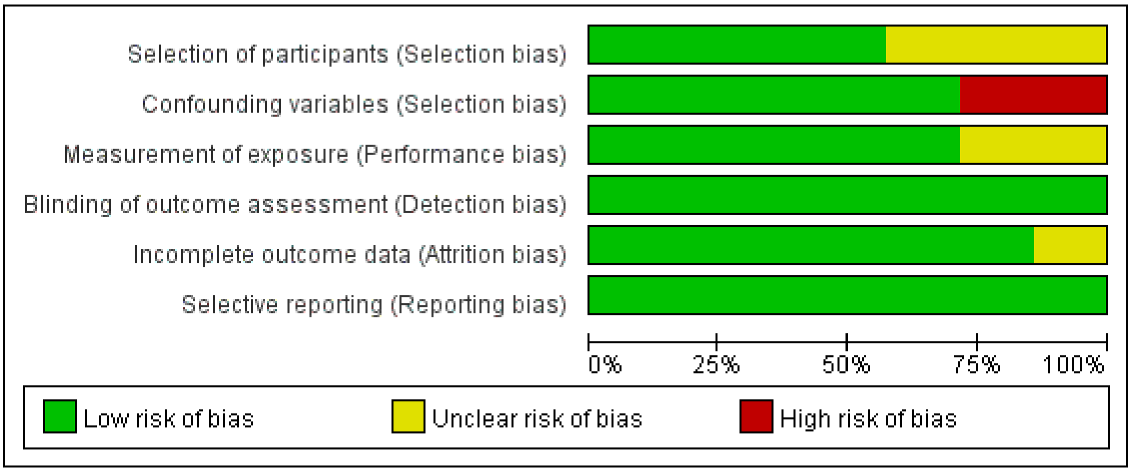

Supplementary Figure 2 Summary of risk of bias for this meta-analysis

	Selection of participants (Selection bias)	Confounding variables (Selection bias)	Measurement of exposure (Performance bias)	Blinding of outcome assessment (Detection bias)	Incomplete outcome data (Attrition bias)	Selective reporting (Reporting bias)
Bluemenstein 2015	?	+	+	+	+	+
Chen 2008	+	+	+	+	+	+
Choi 2015	+	+	+	+	+	+
Chou 2014	?	−	?	+	+	+
Maekawa 2013	+	+	+	+	+	+
Sakamoto 2014	+	−	+	+	?	+
Shin 2011	?	+	?	+	+	+
